# Supplementary material for: Proline rich 11 (PRR11) overexpression amplifies PI3K signaling and promotes antiestrogen resistance in breast cancer
Source: Nat Commun. 2020 Oct 30;11:5488. doi: 10.1038/s41467-020-19291-x (PMC7599336; doi:10.1038/s41467-020-19291-x)
Supplement: Supplementary file 1 — Supplementary Information [file 41467_2020_19291_MOESM1_ESM.pdf]

Supplementary data for

***Proline rich 11* (*PRR11*) overexpression amplifies PI3K signaling and promotes antiestrogen resistance in breast cancer**

Authors: Kyung-min Lee[1], Angel L. Guerrero-Zotano[2], Alberto Servetto[1], Dhivya R. Sudhan[1], Chang-Ching Lin[1], Luigi Formisano[2], Valerie M. Jansen[2], Paula González-Ericsson[3], Melinda E. Sanders[3], Thomas P. Stricker[3], Ganesh Raj[4], Kevin M. Dean[5], Reto Fiolka[5,6], Lewis C. Cantley[7], Ariella B. Hanker[1], Carlos L. Arteaga[1,3,8,*]

[*]Corresponding author. Email: Carlos.Arteaga@UTSouthwestern.edu

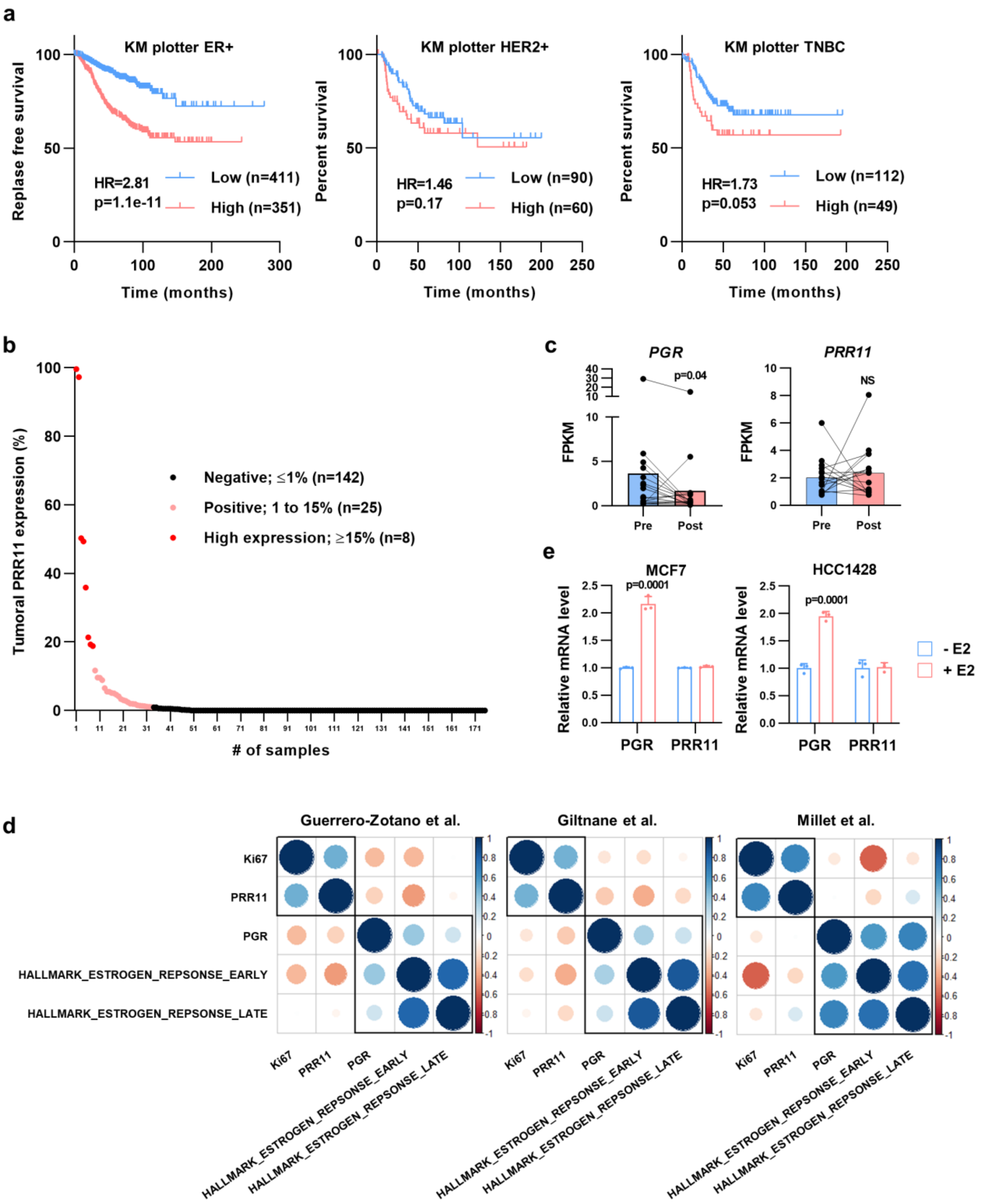

a
KM plotter ER+
Replase free survival
HR=2.81
p=1.1e-11
Low (n=411)
High (n=351)
Time (months)
KM plotter HER2+
Percent survival
HR=1.46
p=0.17
Low (n=90)
High (n=60)
Time (months)
KM plotter TNBC
Percent survival
HR=1.73
p=0.053
Low (n=112)
High (n=49)
Time (months)
b
Tumoral PRR11 expression (%)
Negative; ≤1% (n=142)
Positive; 1 to 15% (n=25)
High expression; ≥15% (n=8)
# of samples
c
PGR
p=0.04
FPKM
Pre
Post
PRR11
NS
FPKM
Pre
Post
e
MCF7
p=0.0001
Relative mRNA level
PGR
PRR11
HCC1428
p=0.0001
Relative mRNA level
PGR
PRR11
- E2
+ E2
d
Guerrero-Zotano et al.
Giltnane et al.
Millet et al.
Ki67
PRR11
PGR
HALLMARK_ESTROGEN_REPSONSE_EARLY
HALLMARK_ESTROGEN_REPSONSE_LATE

Supplementary Fig. 1. ***PRR11*** **overexpression is associated with poor outcome in ER$^+$ breast cancers. a**, Relapse free survival (RFS) of ER$^+$, HER2$^+$ and triple negative breast cancers (n=762, 150 and 161, respectively) with low or high *PRR11* mRNA levels by the auto select best cutoff in Kaplan-Meier Plotter. HR and p were adopted from the Kaplan-Meier Plotter (http://kmplot.com/analysis/). **b**, Distribution of PRR11 positivity in ER$^+$ primary breast tumors (n=175). **c**, Fragments per kilobase of transcript (FPKM) of *PGR* (left) and *PRR11* (right) in paired ER$^+$/HER2$^-$ breast tumors pre- and post-aromatase inhibitor treatment (cohort of Miller et al.: n=16; one-tailed paired t-tests). **d**, Correlation matrices with coefficients in ER$^+$ tumors following neoadjuvant aromatase inhibitor treatment across 3 clinical studies (Pearson correlation). Color scale represents the correlation coefficients. **e**, MCF7 and HCC1428 cells were grown in estrogen (E2)-deprived condition for 24 h then treated ± 1 nM E2 for 24 h. At this time, mRNA was extracted; cDNA was prepared and subjected to RT-qPCR with *PGR*, *PRR11* and *GAPDH* primers. Data represent the mean ± SD of three replicates (two-tailed unpaired t-tests). Source data are provided as a Source data file.

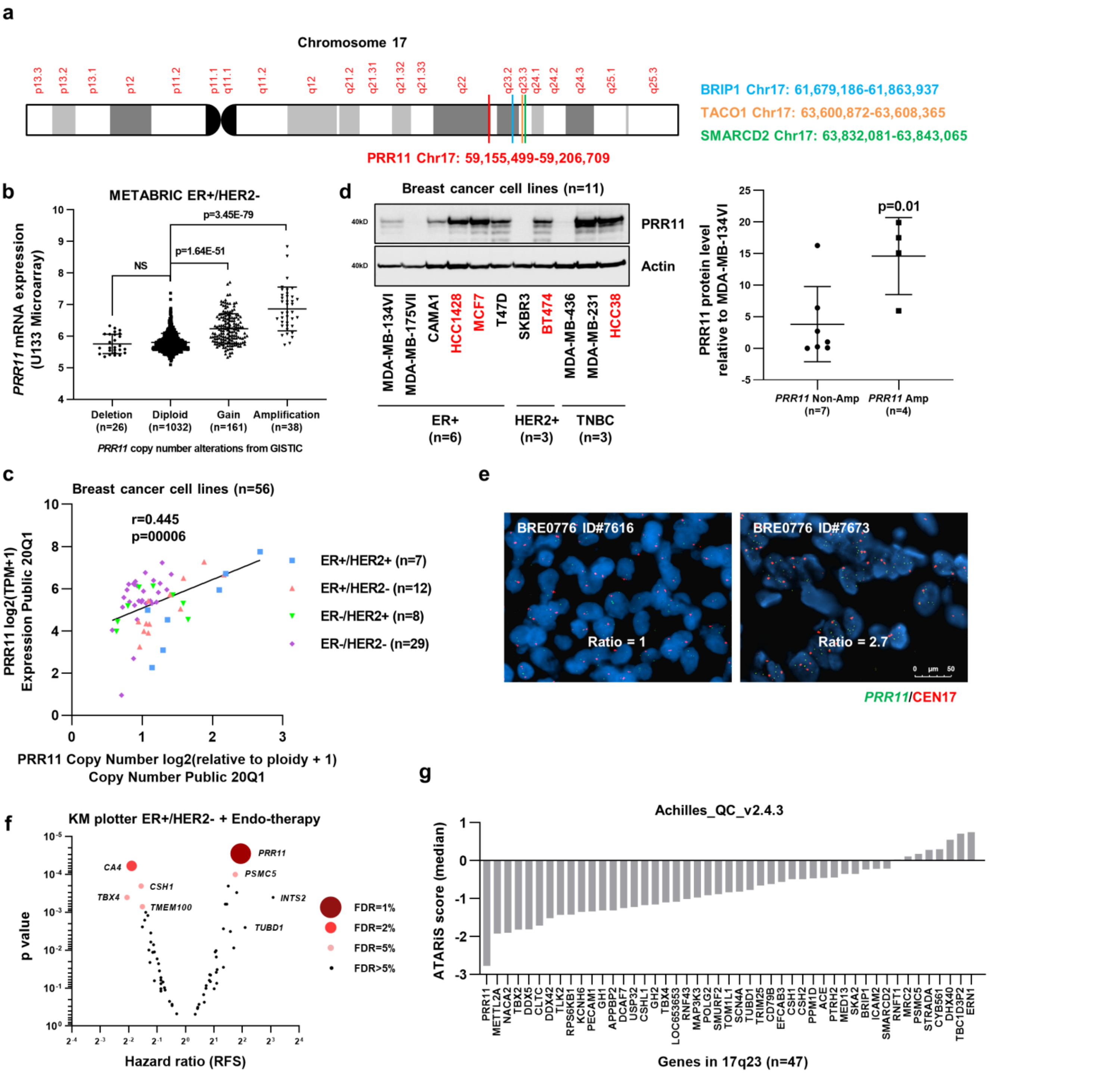

a
Chromosome 17
BRIP1 Chr17: 61,679,186-61,863,937
TACO1 Chr17: 63,600,872-63,608,365
SMARCD2 Chr17: 63,832,081-63,843,065
PRR11 Chr17: 59,155,499-59,206,709
b
METABRIC ER+/HER2-
PRR11 mRNA expression (U133 Microarray)
Deletion (n=26)
Diploid (n=1032)
Gain (n=161)
Amplification (n=38)
PRR11 copy number alterations from GISTIC
NS
p=1.64E-51
p=3.45E-79
c
Breast cancer cell lines (n=56)
r=0.445
p=00006
ER+/HER2+ (n=7)
ER+/HER2- (n=12)
ER-/HER2+ (n=8)
ER-/HER2- (n=29)
PRR11 log2(TPM+1) Expression Public 20Q1
PRR11 Copy Number log2(relative to ploidy + 1) Copy Number Public 20Q1
d
Breast cancer cell lines (n=11)
PRR11
Actin
MDA-MB-134VI
MDA-MB-175VII
CAMA1
HCC1428
MCF7
T47D
SKBR3
BT474
MDA-MB-436
MDA-MB-231
HCC38
ER+ (n=6)
HER2+ (n=3)
TNBC (n=3)
PRR11 protein level relative to MDA-MB-134VI
p=0.01
PRR11 Non-Amp (n=7)
PRR11 Amp (n=4)
e
BRE0776 ID#7616
BRE0776 ID#7673
Ratio = 1
Ratio = 2.7
PRR11/CEN17
f
KM plotter ER+/HER2- + Endo-therapy
p value
Hazard ratio (RFS)
PRR11
PSMC5
CA4
CSH1
TBX4
TMEM100
INTS2
TUBD1
FDR=1%
FDR=2%
FDR=5%
FDR>5%
g
Achilles_QC_v2.4.3
ATARiS score (median)
Genes in 17q23 (n=47)
PRR11 METTL2A NACA2 TBX2 DDX5 CLTC DDX42 TLK2 RPS6KB1 KCNH6 PECAM1 GH1 APPBP2 DCAF7 USP32 CSHL1 GH2 TBX4 LOC653653 RNF43 MAP3K3 POLG2 SMURF2 TOM1L1 SCN4A TUBD1 TRIM25 CD79B EFCAB3 CSH1 CSH2 PPM1D ACE PTRH2 MED13 SKA2 BRIP1 ICAM2 SMARCD2 RNFT1 MRC2 PSMC5 STRADA CYB561 DHX40 TBC1D3P2 ERN1

Supplementary Fig. 2. High *PRR11* mRNA levels are associated with a shorter relapse free survival. a, Colored lines indicate the locus of *PRR11, BRIP1, TACO1 and SMARCD2* in the chromosome 17q. **b**, *PRR11* mRNA levels plotted against *PRR11* copy number alterations (CNAs) ER+/HER- breast tumors in METABRIC (n=1257). Data represent the mean ± SD (two-tailed unpaired t-tests). **c**, *PRR11* copy numbers plotted against *PRR11* mRNA expression in breast cancer cell lines of the DepMap dataset (n=56; Pearson r). **d**, Lysates from breast cancer cell lines were subjected to immunoblot analysis with PRR11 and actin antibodies. *PRR11*-amplified cell lines are highlighted in red. The intensity of PRR11 immunoblot bands was quantified using the Image Lab software (ver. 6.0, BioRad) and then plotted by *PRR11* amplification status (n=7 and 4 for *PRR11* non-amplified and *PRR11* amplified group, respectively). Data represent the mean of PRR11 levels ± SD (two-tailed unpaired t-test). **e**, Representative FISH image from a breast tumor specimen with *PRR11* amplification. Magnification = 100x. **f**, Predicted RFS in patients with $ER^{+}/HER2^{-}$ breast cancer treated with endocrine therapy in the Kaplan-Meier Plotter database as a function of high expression of 67 genes in 17q23. Patients were split by the auto select best cutoff of each gene. Hazard ratio, p value and FDR are plotted; size and color of dots were used to stratify genes based on the FDR. **g**, Scores computed by the Analytic Technique for Assessment of RNAi by Similarity (ATARiS) algorithm that provides an intuitive measure of the effect of gene suppression on cell viability were used for the comparison between genes. Median ATARiS scores of genes in 17q23 amplicon from Project Achilles dataset (Achilles_QC_v2.4.3) were plotted (n=47). Source data are provided as a Source data file.

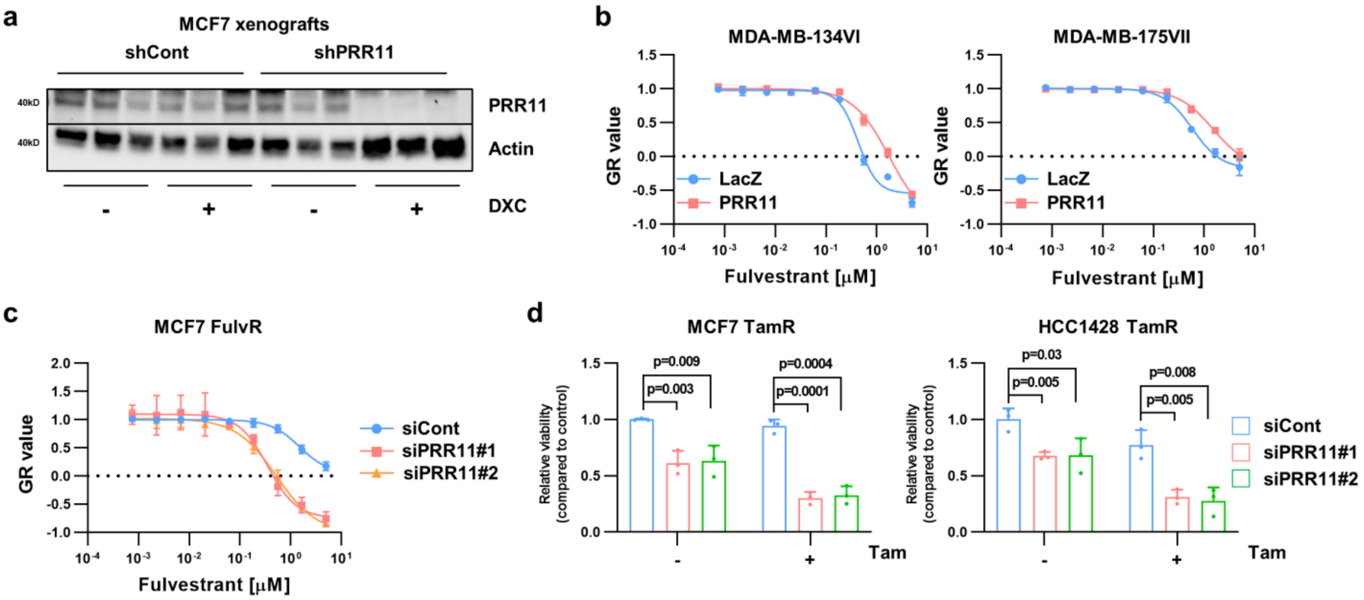

a
MCF7 xenografts
shCont
shPRR11
40kD
PRR11
40kD
Actin
-
+
-
+
DXC
b
MDA-MB-134VI
MDA-MB-175VII
GR value
LacZ
PRR11
Fulvestrant [μM]
c
MCF7 FulvR
GR value
siCont
siPRR11#1
siPRR11#2
Fulvestrant [μM]
d
MCF7 TamR
HCC1428 TamR
Relative viability (compared to control)
p=0.009
p=0.003
p=0.0004
p=0.0001
p=0.03
p=0.005
p=0.008
p=0.005
Tam
siCont
siPRR11#1
siPRR11#2

Supplementary Fig. 3. *PRR11* ablation restores antiestrogens action. a, Whole lysates of MCF7 xenografts harvested at the end of treatment as in Figure 3B were subjected to immunoblot analysis with PRR11 and actin antibodies. **b and c**, Sensitivity to fulvestrant was measured with the GR metrics assay. Cells were treated with multiple doses of fulvestrant for 6 days. Cell numbers on days 0 and 6 were used as the input data. Data represent the mean ± SD of three replicates. **d**, MCF7 TamR and HCC1428 TamR cells were transfected with control or *PRR11* siRNAs. Low density monolayers of cells were treated ± 1 μM tamoxifen for 10 days. Cell monolayers were stained with crystal violet. Each data point represents the mean ± SD three replicates (two-tailed unpaired t-tests). Source data are provided as a Source data file.

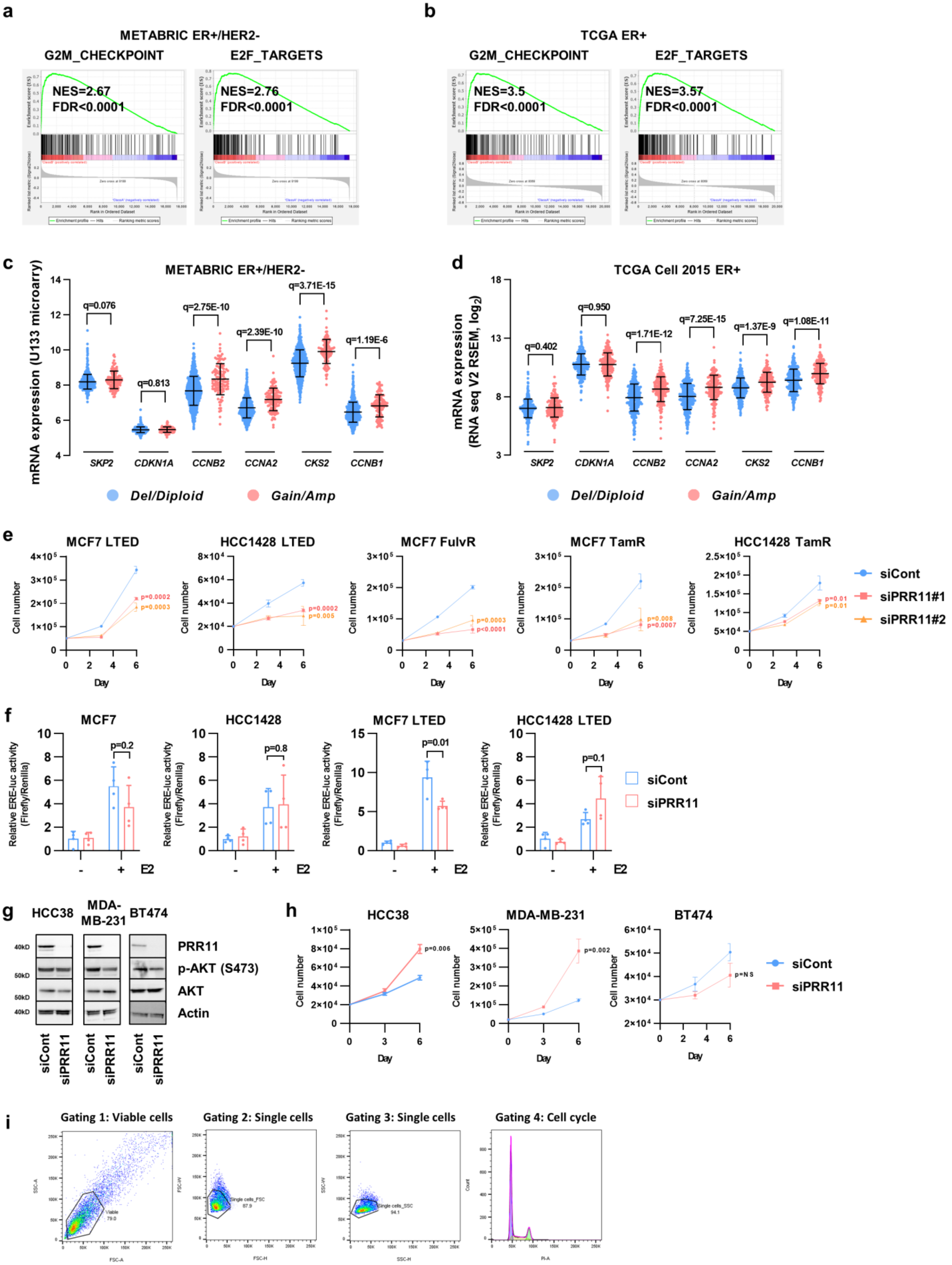
a
METABRIC ER+/HER2-
G2M_CHECKPOINT
E2F_TARGETS
NES=2.67
FDR<0.0001
NES=2.76
FDR<0.0001
b
TCGA ER+
G2M_CHECKPOINT
E2F_TARGETS
NES=3.5
FDR<0.0001
NES=3.57
FDR<0.0001
c
METABRIC ER+/HER2-
mRNA expression (U133 microarry)
q=0.076
q=0.813
q=2.75E-10
q=2.39E-10
q=3.71E-15
q=1.19E-6
SKP2
CDKN1A
CCNB2
CCNA2
CKS2
CCNB1
Del/Diploid
Gain/Amp
d
TCGA Cell 2015 ER+
mRNA expression (RNA seq V2 RSEM, log2)
q=0.402
q=0.950
q=1.71E-12
q=7.25E-15
q=1.37E-9
q=1.08E-11
SKP2
CDKN1A
CCNB2
CCNA2
CKS2
CCNB1
Del/Diploid
Gain/Amp
e
MCF7 LTED
HCC1428 LTED
MCF7 FulvR
MCF7 TamR
HCC1428 TamR
Cell number
Day
p=0.0002
p=0.0003
p=0.0002
p=0.005
p=0.0003
p<0.0001
p=0.008
p=0.0007
p=0.01
p=0.01
siCont
siPRR11#1
siPRR11#2
f
MCF7
HCC1428
MCF7 LTED
HCC1428 LTED
Relative ERE-luc activity (Firefly/Renilla)
p=0.2
p=0.8
p=0.01
p=0.1
E2
siCont
siPRR11
g
HCC38
MDA-MB-231
BT474
40kD
50kD
50kD
40kD
PRR11
p-AKT (S473)
AKT
Actin
siCont
siPRR11
siCont
siPRR11
siCont
siPRR11
h
HCC38
MDA-MB-231
BT474
Cell number
Day
p=0.006
p=0.002
p=N S
siCont
siPRR11
i
Gating 1: Viable cells
Gating 2: Single cells
Gating 3: Single cells
Gating 4: Cell cycle

Supplementary Fig. 4. PRR11 overexpression promotes estrogen-independent cell proliferation. a and b, GSEA of ER+/HER2- tumors in METABRIC (*PRR11* gain/amplification vs deletion/diploid; a) and ER^+ tumors in TCGA (*PRR11* amplification vs no amplification; c); analyses show enrichment in G2M_CHECKPOINT and E2F_TARGETS signatures (NES: Normalized enrichment score). **c and d**, The 6 cell cycle genes downregulated in MCF7 LTED cells upon transfection of *PRR11* siRNA were evaluated in ER+/HER2- tumors in METABRIC [c: *PRR11* gain/amplification (n=113) vs. deletion/diploid (n=1242)] and ER^+ tumors in TCGA [d: *PRR11* gain/amplification (n=231) vs. deletion/diploid (n=362)]; q values were adopted from the cBioPortal. Data represent the mean ± SD. **e**, MCF7 LTED, HCC1428 LTED, MCF7 FulvR, MCF7 TamR and HCC1428 TamR cells were transfected with *PRR11* or control siRNA. Cells were counted every 72 h for 6 days. Data represent the mean ± SD of three replicates (two-tailed unpaired t-tests). **f**, MCF7 parental, HCC1428 parental, MCF7 LTED and HCC1428 LTED cells were transfected with control or *PRR11* siRNA. Cells were then co-transfected with pGLB-MERE and pCMV-Renilla; 24 h post-transfection, cells were switched to estrogen (E2)-free medium, at which time 1 nM estradiol was added for additional 24 h. *Renilla* and firefly luciferase activities were measured using Dual-Luciferase Reporter Assay System. Data represent the mean ± SD of four replicates (two-tailed unpaired t-tests). **g**, Lysates from HCC38, MDA-MB-231 and BT474 cells transfected with control or *PRR11* siRNA for 48 h were subjected to immunoblot analysis with PRR11, p-AKT, AKT and actin antibodies. **h**, HCC38, MDA-MB-231 and BT474 cells were transfected with *PRR11* or control siRNA. Cells were counted every 72 h for 6 days. Data represent the mean ± SD of three replicates (two-tailed unpaired t-tests). **i**, Gating strategy for the cell cycle analyses shown in Fig. 4c-e. Source data are provided as a Source data file.

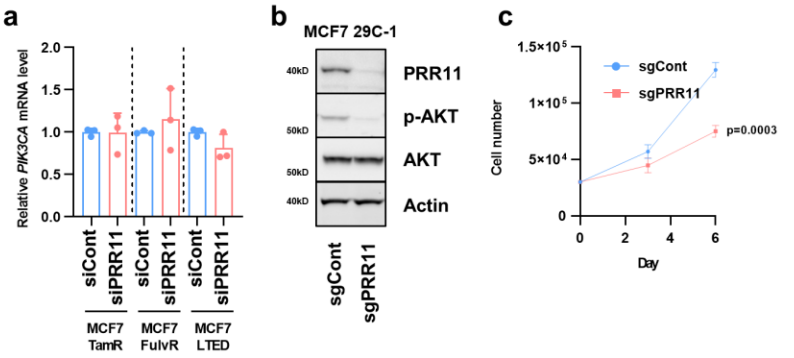
a
b
MCF7 29C-1
c
d
Relative PIK3CA mRNA level
siCont
siPRR11
MCF7 TamR
MCF7 FulvR
MCF7 LTED
PRR11
p-AKT
AKT
Actin
40kD
50kD
50kD
40kD
sgCont
sgPRR11
Cell number
Day
p=0.0003
Relative viability (Compared to sgCont+Fulv)
Relative viability (Compared to sgCont+Tam)
p=0.004
p=0.001
+ Fulv
+ Tam

Supplementary Fig. 5. PRR11 confers antiestrogen resistance in *PIK3CA* wild type cells. a, MCF7 TamR, FulvR and LTED cells were transfected with control or *PRR11* siRNA for 24 h. At this time, mRNA was extracted; cDNA was prepared and subjected to RT-qPCR with *PIK3CA* primers. Data represent the relative mean *PIK3CA* mRNA level ± SD of three replicates. **b**, Lysates of MCF7 29C-1 cells stably transduced with control or *PRR11* sgRNA were subjected to immunoblot analysis with PRR11, p-AKT, AKT and actin antibodies. **c**, MCF7 29C-1 cells transduced with control or *PRR11* sgRNA were counted every 72 h for 6 days. Each data point represents the mean ± SD of three replicates (two-tailed unpaired t-tests). **d**, Low density monolayers of MCF7 29C-1 cells transduced with control or *PRR11* sgRNA were treated ± 1 μM fulvestrant (left) or 1 μM tamoxifen (right) for 10 days. Cell monolayers were stained with crystal violet. Each data point represents the mean ± SD of three replicates (two-tailed unpaired t-tests). Source data are provided as a Source data file.

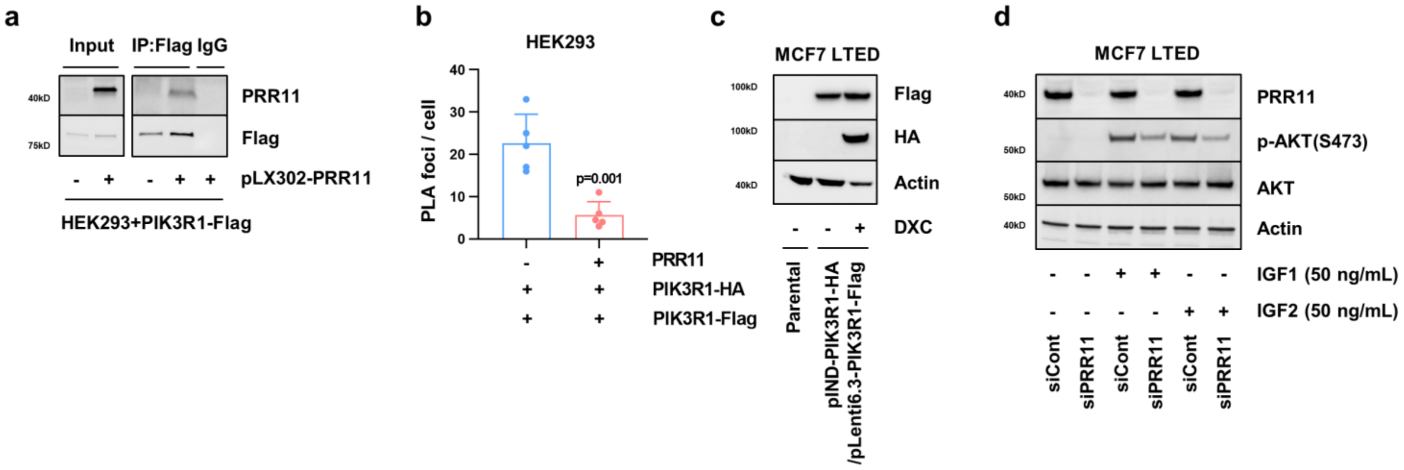
a
Input
IP:Flag
IgG
40kD
75kD
PRR11
Flag
pLX302-PRR11
HEK293+PIK3R1-Flag
b
HEK293
PLA foci / cell
p=0.001
PRR11
PIK3R1-HA
PIK3R1-Flag
c
MCF7 LTED
100kD
40kD
Flag
HA
Actin
DXC
Parental
pIND-PIK3R1-HA
/pLenti6.3-PIK3R1-Flag
d
MCF7 LTED
PRR11
p-AKT(S473)
AKT
Actin
50kD
IGF1 (50 ng/mL)
IGF2 (50 ng/mL)
siCont
siPRR11

Supplementary Fig. 6. PRR11 enhances IGF1/2-induced pAKT. a, Lysates of HEK293 cells transduced with pLenti7.3-*PIK3R1*-Flag and pLX302-*PRR11*-V5 were immunoprecipitated with Flag or IgG antibodies; immune complexes were then subjected to immunoblot analysis with V5 and Flag antibodies. **b**, HEK293 cells were co-transduced with pLenti7.3-*PIK3R1*-Flag, -*PIK3R1*-HA, and pLX302-*PRR11*-V5 and then subjected to PLA with HA and Flag antibodies. Each bar represents the average number of PLA foci per cell (indicative of p85 dimers) of 5 images ± SD (two-tailed unpaired t-tests). **c**, MCF7 LTED cells were co-transduced with pLenti6.3-*PIK3R1*-Flag and pIND-*PIK3R1*-HA. Where indicated, cells were treated ± 0.2 μg/mL doxycycline (DXC) for 48 h and then subjected to immunoblot analysis with Flag, HA and actin antibodies. **d**, MCF7 LTED cells were transfected with control or *PRR11* siRNA for 24 h. Cells were then treated with 50 ng/mL IGF1 or 50 ng/mL IGF2 for 10 min. Cell lysates were subjected to immunoblot analysis with PRR11, p-AKT, AKT, and actin antibodies. Source data are provided as a Source data file.

a

Motif Scan Result	Position	N-Score	E-value
prf:PRO_RICH; *Proline-rich region profile*	174-202	15.645	4.8e-09
pfam_fs:zf-ZPR1; *ZPR1 zinc-finger domain*	139-159	10.795	0.00034

b

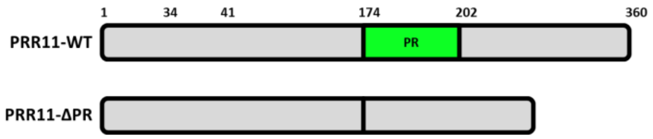

c

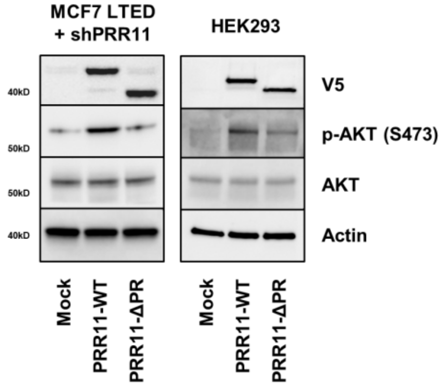

Supplementary Fig. 7. PRR11 mutant lacking PR motif fails to activate AKT. a, Motifs in the PRR11 protein were determined with the motif scan (https://myhits.isb-sib.ch/cgi-bin/motif_scan). **b**, A PRR11 mutant lacking PR motif (*PRR11*-ΔPR) was designed based on the motif prediction in S6A. **c**, MCF7 LTED cells that had been stably transduced with shRNA targeting the 3'UTR of *PRR11* were re-transduced with pLX304-*PRR11* wild type (WT) or pLX304-*PRR11* ΔPR. HEK293 cells were transduced with pLX302-*PRR11* WT or pLX302-*PRR11* ΔPR for 48 h. Cell lysates were subjected to immunoblot analysis with V5, p-AKT, AKT and actin antibodies. Source data are provided as a Source data file.

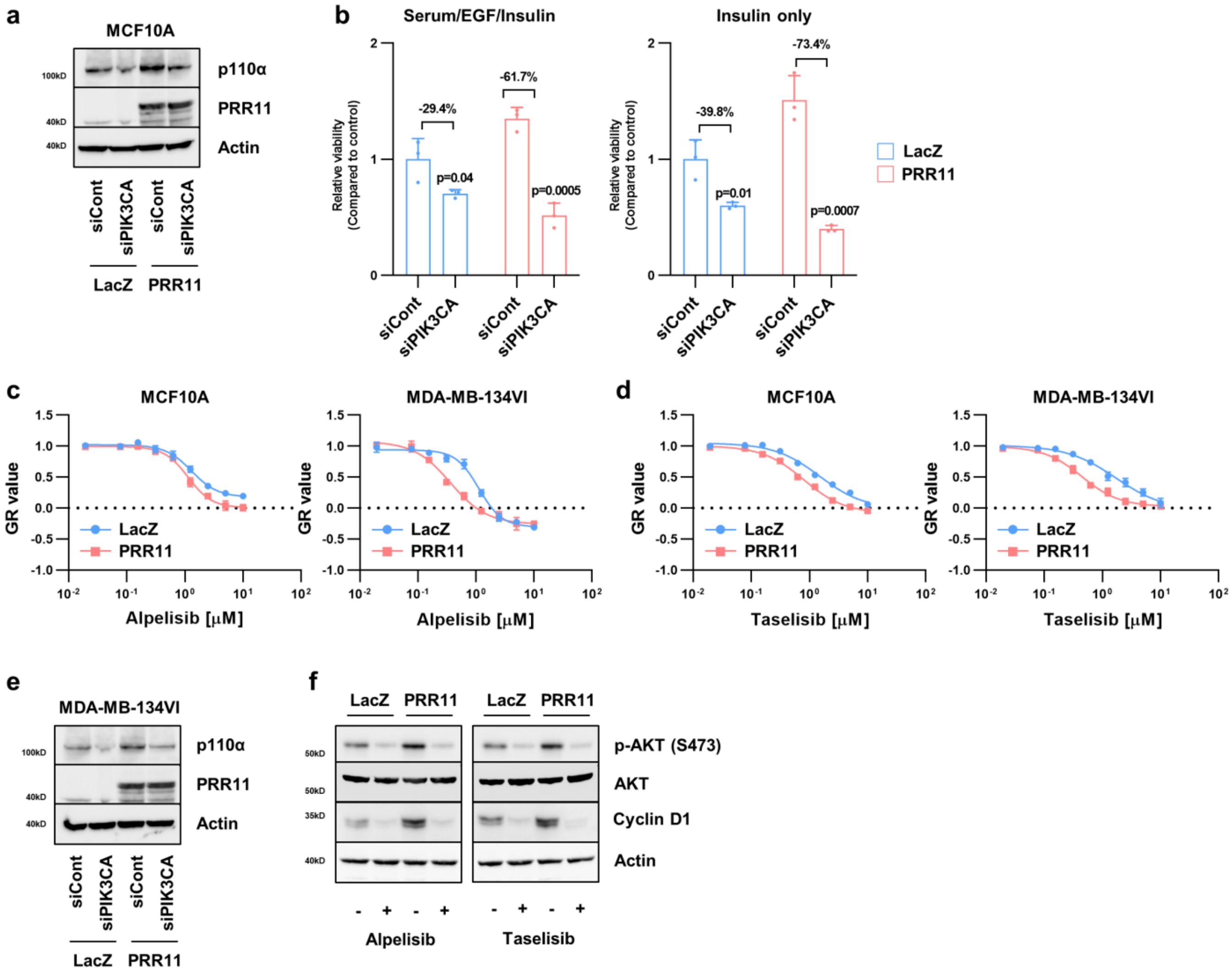
a
MCF10A
100kD
40kD
40kD
p110α
PRR11
Actin
siCont
siPIK3CA
siCont
siPIK3CA
LacZ
PRR11
b
Serum/EGF/Insulin
Insulin only
Relative viability (Compared to control)
-29.4%
-61.7%
p=0.04
p=0.0005
-39.8%
-73.4%
p=0.01
p=0.0007
siCont
siPIK3CA
siCont
siPIK3CA
LacZ
PRR11
c
MCF10A
MDA-MB-134VI
GR value
Alpelisib [μM]
LacZ
PRR11
d
MCF10A
MDA-MB-134VI
GR value
Taselisib [μM]
LacZ
PRR11
e
MDA-MB-134VI
100kD
40kD
40kD
p110α
PRR11
Actin
siCont
siPIK3CA
siCont
siPIK3CA
LacZ
PRR11
f
LacZ
PRR11
LacZ
PRR11
50kD
50kD
35kD
40kD
p-AKT (S473)
AKT
Cyclin D1
Actin
Alpelisib
Taselisib

Supplementary Fig. 8. *PIK3CA* ablation abrogates PRR11-mediated estrogen-independent growth. a, MCF10A stably transduced with pLX302-*LacZ* or pLX302-*PRR11* cells were transfected with control or *PIK3CA* siRNA for 48 h. Lysates were subjected to immunoblot analysis with p110α, PRR11, and actin antibodies. **b**, MCF10A pLX302-*LacZ* and -*PRR11* cells were transfected with control or *PIK3CA* siRNA and then grown in full media (DMEM/F12 with 5% horse serum, 20 ng/mL EGF, 10 µg/mL insulin; left) or estrogen-free, low serum media (DMEM/F12 with 1% charcoal dextran-stripped serum, 10 µg/mL insulin; right). After 10 days, monolayers were stained with crystal violet. Data represent the mean ± SD of three replicates (two-tailed unpaired t-tests). **c and d**, Sensitivity to alpelisib (c) and taselisib (d) was measuredwith the GR metrics assay. Cells were treated with multiple doses of alpelisib and taselisib for 6 days. Cell numbers on days 0 and 6 were used as the input data. Data represent the mean ± SD of three replicates. **e**, MDA-MB-134VI pLX302-*LacZ* and -*PRR11* cells were transfected with control or *PIK3CA* siRNA for 48 h. Cell lysates were then subjected to immunoblot analysis with p110α, PRR11 and actin antibodies. **f,** MDA-MB-134VI pLX302-*LacZ* cells and -*PRR11* cells were treated ± 1 µM alpelisib or ± 1 µM taselisib for 24 h. Cell lysates were subjected to immunoblot analysis with p-AKT, AKT, cyclin D1 and actin antibodies. Source data are provided as a Source data file.

Supplementary Table 1. Summary of clinical studies with neoadjuvant aromatase inhibitors

Cohort	Patients (n)	Receptor status	Menopause Status	Treatment (Time)	Ref	Stage
Guerrero-Zotano et al	58	ER+/HER2-	Postmenopausal	Neoadjuvant letrozole (Median of 7.2 months)	17	II-III
Giltnane et al	45	ER+/HER2-	Postmenopausal	Neoadjuvant letrozole (10-21 days)	14	I-III
Miller et al	18	ER+	Postmenopausal	Neoadjuvant aromatase inhibitors (4 months)	22	II-III

Supplementary Table 2. List of genes in the 17q23 locus

Symbol(↓) ↓	Location(↓)	Description	GoldenPath hg38 (Mb)(↓)
LINC01476	17q22-q23.1	long intergenic non-protein coding RNA 1476	59430.869
MRC2	17q23	mannose receptor C type 2	62627.401
TLK2	17q23	tousled like kinase 2	62479.025
CA4	17q23.1	carbonic anhydrase 4	60149.936
CLTC	17q23.1	clathrin heavy chain	59619.689
DHX40	17q23.1	DEAH-box helicase 40	59565.525
HEATR6	17q23.1	HEAT repeat containing 6	60043.191
LOC100996660	17q23.1	uncharacterized LOC100996660	60134.622
LOC101927755	17q23.1	uncharacterized LOC101927755	59965.096
LOC105371849	17q23.1	uncharacterized LOC105371849	60079.309
LOC653653	17q23.1	adaptor related protein complex 1 sigma 2 subunit pseudogene	60101.76
MIR21	17q23.1	microRNA 21	59841.266
MIR4737	17q23.1	microRNA 4737	60043.025
PTRH2	17q23.1	peptidyl-tRNA hydrolase 2	59697.306
RNFT1	17q23.1	ring finger protein, transmembrane 1	59952.362
RPS6KB1	17q23.1	ribosomal protein S6 kinase B1	59893.046
TBC1D3P1-DHX40P1	17q23.1	TBC1D3P1-DHX40P1 readthrough, transcribed pseudogene	59962.363
TMEM100	17q23.1	transmembrane protein 100	55719.627
TRIM25	17q23.1	tripartite motif containing 25	56887.909
TUBD1	17q23.1	tubulin delta 1	59859.48
VMP1	17q23.1	vacuole membrane protein 1	59707.465
WFDC21P	17q23.1	WAP four-disulfide core domain 21, pseudogene	60083.566
APPBP2	17q23.2	amyloid beta precursor protein binding protein 2	60443.149
BCAS3	17q23.2	BCAS3, microtubule associated cell migration factor	60677.811
BRIP1	17q23.2	BRCA1 interacting protein C-terminal helicase 1	61679.186
C17orf64	17q23.2	chromosome 17 open reading frame 64	60422.504
C17orf82	17q23.2	chromosome 17 open reading frame 82	61411.751
EFCAB3	17q23.2	EF-hand calcium binding domain 3	62370.218
INTS2	17q23.2	integrator complex subunit 2	61865.367
LINC01999	17q23.2	long intergenic non-protein coding RNA 1999	60564.546
LOC101927855	17q23.2	uncharacterized LOC101927855	61361.668
LOC101927877	17q23.2	uncharacterized LOC101927877	62808.502
MARCH10	17q23.2	membrane associated ring-CH-type finger 10	62701.314
MED13	17q23.2	mediator complex subunit 13	61942.605
METTL2A	17q23.2	methyltransferase like 2A	62423.885
MIR633	17q23.2	microRNA 633	62944.215
NACA2	17q23.2	nascent polypeptide associated complex alpha subunit 2	61590.433
POLRMTP1	17q23.2	RNA polymerase mitochondrial pseudogene 1	62137.17
PRR11	17q23.2	proline rich 11	59155.499
RNF43	17q23.2	ring finger protein 43	58352.5
RPL12P38	17q23.2	ribosomal protein L12 pseudogene 38	60433.678
SCARNA20	17q23.2	small Cajal body-specific RNA 20	60231.516
SKA2	17q23.2	spindle and kinetochore associated complex subunit 2	59109.947
TBC1D3P2	17q23.2	TBC1 domain family member 3 pseudogene 2	62264.706

TBX2	17q23.2	T-box 2	61399.896
TBX2-AS1	17q23.2	TBX2 antisense RNA 1	61393.456
TBX4	17q23.2	T-box 4	61452.418
TOM1L1	17q23.2	target of myb1 like 1 membrane trafficking protein	54900.691
TANC2	17q23.2-q23.3	tetratricopeptide repeat, ankyrin repeat and coiled-coil containing 2	63009.537
ACE	17q23.3	angiotensin I converting enzyme	63477.061
CCDC47	17q23.3	coiled-coil domain containing 47	63745.25
CD79B	17q23.3	CD79b molecule	63928.736
CEP95	17q23.3	centrosomal protein 95	64506.736
CSH1	17q23.3	chorionic somatomammotropin hormone 1	63894.908
CSH2	17q23.3	chorionic somatomammotropin hormone 2	63872.012
CSHL1	17q23.3	chorionic somatomammotropin hormone like 1	63909.597
CYB561	17q23.3	cytochrome b561	63432.304
DCAF7	17q23.3	DDB1 and CUL4 associated factor 7	63550.435
DDX42	17q23.3	DEAD-box helicase 42	63774.189
DDX5	17q23.3	DEAD-box helicase 5	64498.254
ERN1	17q23.3	endoplasmic reticulum to nucleus signaling 1	64043.03
FTSJ3	17q23.3	FtsJ homolog 3	63819.433
GH1	17q23.3	growth hormone 1	63917.193
GH2	17q23.3	growth hormone 2	63880.212
ICAM2	17q23.3	intercellular adhesion molecule 2	64002.595
KCNH6	17q23.3	potassium voltage-gated channel subfamily H member 6	63523.334
LIMD2	17q23.3	LIM domain containing 2	63695.889
LOC729683	17q23.3	uncharacterized LOC729683	63700.338
MAP3K3	17q23.3	mitogen-activated protein kinase kinase kinase 3	63622.425
MILR1	17q23.3	mast cell immunoglobulin like receptor 1	64449.115
MIR1273E	17q23.3	microRNA 1273e	64425.069
MIR3064	17q23.3	microRNA 3064	64500.774
MIR5047	17q23.3	microRNA 5047	64501.214
PECAM1	17q23.3	platelet and endothelial cell adhesion molecule 1	64319.416
POLG2	17q23.3	DNA polymerase gamma 2, accessory subunit	64477.785
PPM1D	17q23.3	protein phosphatase, Mg2+/Mn2+ dependent 1D	60600.183
PRR29	17q23.3	proline rich 29	63998.351
PRR29-AS1	17q23.3	PRR29 antisense RNA 1	63996.071
PSMC5	17q23.3	proteasome 26S subunit, ATPase 5	63827.684
SCN4A	17q23.3	sodium voltage-gated channel alpha subunit 4	63938.554
SMARCD2	17q23.3	SWI/SNF related, matrix associated, actin dependent regulator of chromatin, subfamily d, member 2	63832.081
SNHG25	17q23.3	small nucleolar RNA host gene 25	64145.97
SNORA50C	17q23.3	small nucleolar RNA, H/ACA box 50C	64146.339
SNORD104	17q23.3	small nucleolar RNA, C/D box 104	64146.078
STRADA	17q23.3	STE20-related kinase adaptor alpha	63702.832
TACO1	17q23.3	translational activator of cytochrome c oxidase I	63600.872
TCAM1P	17q23.3	testicular cell adhesion molecule 1, pseudogene	63857.016
TEX2	17q23.3	testis expressed 2	64147.433
USP32	17q23.3	ubiquitin specific peptidase 32	60177.33
SMURF2	17q23.3-q24.1	SMAD specific E3 ubiquitin protein ligase 2	64544.617

Supplementary Table 3. Correlation between Ki67+ % and mRNA expression levels of 17q23 region genes in the cohort of Guerrero-Zotano

Gene	Pearson r	95% confidence interval	R squared	P (two-tailed)	P value summary	Significant? (alpha = 0.05)	Number of XY Pairs
PRR11	0.4838	0.2577 to 0.6596	0.234	0.0001	***	Yes	58
BRIP1	0.4278	0.1905 to 0.6178	0.183	0.0008	***	Yes	58
CLTC	0.3802	0.1352 to 0.5814	0.1446	0.0032	**	Yes	58
SKA2	0.3713	0.125 to 0.5745	0.1379	0.0041	**	Yes	58
METTL2A	0.3486	0.09922 to 0.5567	0.1215	0.0073	**	Yes	58
DCAF7	0.3443	0.09439 to 0.5534	0.1185	0.0081	**	Yes	58
MED13	0.3073	0.05319 to 0.524	0.09441	0.019	*	Yes	58
PSMC5	0.307	0.05291 to 0.5238	0.09426	0.0191	*	Yes	58
SMARCD2	0.3031	0.04858 to 0.5206	0.09185	0.0207	*	Yes	58
CCDC47	0.2801	0.0235 to 0.5021	0.07845	0.0332	*	Yes	58
FTSJ3	0.2666	0.008945 to 0.4911	0.07109	0.0431	*	Yes	58
DDX42	0.2593	0.001061 to 0.4851	0.06723	0.0494	*	Yes	58
TRIM25	0.254	-0.004616 to 0.4807	0.06451	0.0544	ns	No	58
TLK2	0.2377	-0.02191 to 0.4673	0.05652	0.0723	ns	No	58
DDX5	0.2375	-0.02219 to 0.4671	0.05639	0.0727	ns	No	58
ERN1	0.2014	-0.06001 to 0.437	0.04056	0.1295	ns	No	58
SMURF2	0.1982	-0.06337 to 0.4342	0.03927	0.1359	ns	No	58
CYB561	0.1762	-0.08599 to 0.4156	0.03105	0.1858	ns	No	58
TEX2	0.1695	-0.09285 to 0.4099	0.02873	0.2033	ns	No	58
DHX40	0.1649	-0.09751 to 0.4059	0.02721	0.216	ns	No	58
APPBP2	0.1538	-0.1088 to 0.3963	0.02365	0.2491	ns	No	58
INTS2	0.1503	-0.1123 to 0.3933	0.02259	0.2601	ns	No	58
PTRH2	0.1427	-0.12 to 0.3868	0.02037	0.2851	ns	No	58
CEP95	0.1299	-0.1329 to 0.3756	0.01686	0.3312	ns	No	58
RPS6KB1	0.1242	-0.1385 to 0.3706	0.01542	0.353	ns	No	58
TANC2	0.1014	-0.1611 to 0.3505	0.01029	0.4487	ns	No	58
TOM1L1	0.1013	-0.1612 to 0.3505	0.01027	0.4491	ns	No	58
HEATR6	0.09616	-0.1663 to 0.3459	0.009246	0.4727	ns	No	58
PPM1D	0.08791	-0.1743 to 0.3385	0.007728	0.5117	ns	No	58
RNF43	0.08049	-0.1816 to 0.3319	0.006479	0.5481	ns	No	58
USP32	0.05665	-0.2046 to 0.3104	0.003209	0.6728	ns	No	58
TUBD1	0.05613	-0.2051 to 0.3099	0.003151	0.6756	ns	No	58
MILR1	0.05585	-0.2054 to 0.3097	0.003119	0.6771	ns	No	58
LIMD2	0.05225	-0.2089 to 0.3064	0.00273	0.6969	ns	No	58
TACO1	0.03084	-0.2293 to 0.2868	0.0009509	0.8183	ns	No	58
SCARNA20	0.02189	-0.2378 to 0.2786	0.0004791	0.8704	ns	No	58
RNFT1	-0.002899	-0.261 to 0.2556	0.000008407	0.9828	ns	No	58
ACE	-0.01545	-0.2727 to 0.2438	0.0002386	0.9084	ns	No	58
POLG2	-0.03365	-0.2894 to 0.2266	0.001132	0.802	ns	No	58
PECAM1	-0.05261	-0.3067 to 0.2085	0.002767	0.6949	ns	No	58
SNORD104	-0.1143	-0.3619 to 0.1484	0.01307	0.3929	ns	No	58
MAP3K3	-0.1153	-0.3628 to 0.1474	0.01329	0.3889	ns	No	58
BCAS3	-0.1213	-0.3681 to 0.1414	0.01473	0.3642	ns	No	58
VMP1	-0.1247	-0.371 to 0.1381	0.01554	0.3511	ns	No	58

ICAM2	-0.1335	-0.3787 to 0.1293	0.01781	0.3179	ns	No	58
RPL12P38	-0.1585	-0.4004 to 0.104	0.02514	0.2346	ns	No	58
MRC2	-0.1813	-0.42 to 0.08073	0.03289	0.1731	ns	No	58
STRADA	-0.2799	-0.5019 to -0.02326	0.07833	0.0334	*	Yes	58
TMEM100	-0.3133	-0.5288 to -0.05981	0.09814	0.0166	*	Yes	58
TBX2	-0.3253	-0.5383 to -0.07312	0.1058	0.0127	*	Yes	58
SCN4A	-0.3342	-0.5454 to -0.0831	0.1117	0.0103	*	Yes	58

Supplementary Table 4. Correlation between Ki67+ % and mRNA expression levels of 17q23 region genes in the cohort of Giltnane

Gene	Pearson r	95% confidence interval	R squared	P (two-tailed)	P value summary	Significant? (alpha = 0.05)	Number of XY Pairs
PRR11	0.4703	0.2051 to 0.6712	0.2212	0.0011	**	Yes	45
SMARCD2	0.4532	0.1841 to 0.659	0.2054	0.0018	**	Yes	45
TACO1	0.4268	0.1524 to 0.6402	0.1822	0.0035	**	Yes	45
CLTC	0.3813	0.09885 to 0.6069	0.1454	0.0098	**	Yes	45
SKA2	0.3432	0.05517 to 0.5784	0.1178	0.021	*	Yes	45
USP32	0.3123	0.02069 to 0.555	0.09755	0.0367	*	Yes	45
CYB561	0.2718	-0.02366 to 0.5235	0.07385	0.0709	ns	No	45
DCAF7	0.2671	-0.02871 to 0.5199	0.07133	0.0761	ns	No	45
FTSJ3	0.2406	-0.05691 to 0.4989	0.05791	0.1113	ns	No	45
PTRH2	0.2386	-0.05907 to 0.4973	0.05693	0.1145	ns	No	45
TLK2	0.2343	-0.06362 to 0.4939	0.05489	0.1214	ns	No	45
DDX42	0.2276	-0.07067 to 0.4885	0.05179	0.1327	ns	No	45
INTS2	0.2241	-0.07431 to 0.4857	0.05023	0.1389	ns	No	45
TRIM25	0.2139	-0.08499 to 0.4774	0.04574	0.1583	ns	No	45
BRIP1	0.2075	-0.09165 to 0.4722	0.04304	0.1715	ns	No	45
EFCAB3	-0.1937	-0.461 to 0.1059	0.0375	0.2024	ns	No	45
TMEM100	-0.1926	-0.4602 to 0.1069	0.03711	0.2049	ns	No	45
LIMD2	0.1913	-0.1083 to 0.4591	0.0366	0.2081	ns	No	45
STRADA	0.1809	-0.1189 to 0.4506	0.03274	0.2342	ns	No	45
HEATR6	0.1727	-0.1272 to 0.4438	0.02984	0.2565	ns	No	45
POLG2	0.1686	-0.1315 to 0.4403	0.02842	0.2683	ns	No	45
C17orf82	0.1569	-0.1433 to 0.4306	0.0246	0.3035	ns	No	45
RNF43	0.149	-0.1511 to 0.424	0.02221	0.3285	ns	No	45
MRC2	-0.1457	-0.4212 to 0.1544	0.02123	0.3396	ns	No	45
C17orf64	0.1409	-0.1592 to 0.4172	0.01985	0.356	ns	No	45
ACE	-0.1276	-0.4059 to 0.1724	0.01629	0.4035	ns	No	45
KCNH6	-0.1232	-0.4022 to 0.1767	0.01519	0.4199	ns	No	45
MED13	0.1203	-0.1795 to 0.3998	0.01448	0.431	ns	No	45
PSMC5	0.1196	-0.1803 to 0.3991	0.0143	0.4339	ns	No	45
NACA2	-0.1156	-0.3957 to 0.1841	0.01337	0.4494	ns	No	45
SCN4A	-0.1098	-0.3907 to 0.1899	0.01205	0.4729	ns	No	45
CCDC47	0.1075	-0.192 to 0.3888	0.01157	0.4819	ns	No	45
DHX40	0.1042	-0.1953 to 0.386	0.01087	0.4956	ns	No	45
CEP95	0.09952	-0.1999 to 0.3819	0.009903	0.5154	ns	No	45
MAP3K3	-0.07644	-0.3619 to 0.2221	0.005844	0.6177	ns	No	45
TEX2	-0.07642	-0.3618 to 0.2221	0.00584	0.6178	ns	No	45
ICAM2	-0.07424	-0.3599 to 0.2242	0.005512	0.6279	ns	No	45
VMP1	0.07061	-0.2276 to 0.3568	0.004986	0.6448	ns	No	45
TUBD1	-0.07029	-0.3565 to 0.2279	0.004941	0.6464	ns	No	45
METTL2A	0.06956	-0.2286 to 0.3558	0.004839	0.6498	ns	No	45
APPBP2	0.06838	-0.2298 to 0.3548	0.004675	0.6554	ns	No	45
SMURF2	0.06837	-0.2298 to 0.3548	0.004675	0.6554	ns	No	45
ERN1	0.06586	-0.2322 to 0.3526	0.004337	0.6673	ns	No	45
GH2	-0.06339	-0.3504 to 0.2345	0.004018	0.6791	ns	No	45

GH1	-0.06325	-0.3503 to 0.2346	0.004	0.6798	ns	No	45
TBX4	0.06285	-0.235 to 0.3499	0.00395	0.6817	ns	No	45
RNFT1	-0.05569	-0.3436 to 0.2418	0.003102	0.7163	ns	No	45
TANC2	-0.04922	-0.3379 to 0.2479	0.002423	0.7481	ns	No	45
CA4	-0.04059	-0.3302 to 0.256	0.001648	0.7912	ns	No	45
CD79B	-0.04022	-0.3299 to 0.2563	0.001618	0.7931	ns	No	45
TBX2	-0.03974	-0.3294 to 0.2568	0.001579	0.7955	ns	No	45
CSH2	0.03556	-0.2607 to 0.3257	0.001265	0.8166	ns	No	45
CSHL1	-0.03218	-0.3227 to 0.2638	0.001035	0.8338	ns	No	45
BCAS3	0.02889	-0.2669 to 0.3197	0.000835	0.8506	ns	No	45
PPM1D	-0.0267	-0.3177 to 0.2689	0.000713	0.8618	ns	No	45
CSH1	-0.02466	-0.3159 to 0.2708	0.000608	0.8723	ns	No	45
DDX5	-0.01981	-0.3115 to 0.2753	0.000393	0.8972	ns	No	45
TOM1L1	-0.01426	-0.3065 to 0.2804	0.000203	0.9259	ns	No	45

Supplementary Table 5. Correlation between Ki67+ % and mRNA expression levels of 17q23 region genes in the cohort of Miller

Gene	Pearson r	95% confidence interval	R squared	P (two-tailed)	P value summary	Significant? (alpha = 0.05)	Number of XY Pairs
PRR11	0.6506	0.264 to 0.8571	0.4233	0.0035	**	Yes	18
STRADA	-0.5839	-0.8257 to -0.1609	0.3409	0.011	*	Yes	18
HEATR6	-0.5553	-0.8117 to -0.1194	0.3084	0.0167	*	Yes	18
RNF43	-0.487	-0.7772 to -0.02603	0.2372	0.0404	*	Yes	18
RNFT1	-0.4399	-0.7523 to 0.03397	0.1935	0.0677	ns	No	18
TBX2	-0.4397	-0.7521 to 0.03424	0.1933	0.0679	ns	No	18
TUBD1	-0.3908	-0.7254 to 0.09299	0.1528	0.1088	ns	No	18
TOM1L1	-0.3894	-0.7246 to 0.09465	0.1517	0.1102	ns	No	18
PPM1D	-0.3531	-0.704 to 0.1362	0.1247	0.1506	ns	No	18
APPBP2	-0.337	-0.6946 to 0.1541	0.1136	0.1714	ns	No	18
TMEM100	-0.3255	-0.6878 to 0.1667	0.1059	0.1875	ns	No	18
CEP95	-0.324	-0.6869 to 0.1684	0.1049	0.1897	ns	No	18
USP32	-0.3102	-0.6788 to 0.1832	0.09623	0.2103	ns	No	18
MAP3K3	-0.3013	-0.6735 to 0.1926	0.0908	0.2243	ns	No	18
ERN1	0.3005	-0.1935 to 0.673	0.09031	0.2256	ns	No	18
DHX40	-0.2595	-0.6479 to 0.236	0.06733	0.2984	ns	No	18
CD79B	0.256	-0.2395 to 0.6457	0.06555	0.3052	ns	No	18
MED13	-0.2381	-0.6345 to 0.2574	0.05671	0.3413	ns	No	18
LIMD2	0.2324	-0.263 to 0.6308	0.05401	0.3534	ns	No	18
SKA2	0.227	-0.2683 to 0.6274	0.05154	0.365	ns	No	18
TEX2	0.2133	-0.2816 to 0.6186	0.04551	0.3953	ns	No	18
MILR1	0.2094	-0.2853 to 0.6161	0.04387	0.4042	ns	No	18
INTS2	-0.1793	-0.5963 to 0.3138	0.03216	0.4765	ns	No	18
ACE	-0.1684	-0.589 to 0.3239	0.02837	0.5041	ns	No	18
ICAM2	0.153	-0.338 to 0.5785	0.02341	0.5445	ns	No	18
BCAS3	-0.1437	-0.5722 to 0.3464	0.02065	0.5694	ns	No	18
CCDC47	-0.139	-0.5689 to 0.3506	0.01932	0.5823	ns	No	18
FTSJ3	0.1374	-0.3521 to 0.5678	0.01887	0.5868	ns	No	18
TACO1	-0.1357	-0.5667 to 0.3535	0.01843	0.5912	ns	No	18
DDX5	-0.1329	-0.5647 to 0.3561	0.01766	0.5991	ns	No	18
SMARCD2	0.1327	-0.3563 to 0.5646	0.0176	0.5997	ns	No	18
RPS6KB1	-0.1188	-0.5549 to 0.3685	0.01411	0.6388	ns	No	18
TANC2	0.1176	-0.3696 to 0.554	0.01383	0.6421	ns	No	18
VMP1	-0.1127	-0.5506 to 0.3738	0.0127	0.6561	ns	No	18
SNORD104	0.1021	-0.383 to 0.5431	0.01043	0.6868	ns	No	18
PSMC5	-0.1008	-0.5422 to 0.3841	0.01017	0.6905	ns	No	18
MRC2	0.07226	-0.4084 to 0.5215	0.005222	0.7757	ns	No	18
POLG2	-0.06693	-0.5176 to 0.4128	0.00448	0.7919	ns	No	18
DCAF7	-0.06532	-0.5293 to 0.4288	0.004267	0.8033	ns	No	17
DDX42	0.05803	-0.4202 to 0.5111	0.003367	0.8191	ns	No	18
SMURF2	0.05504	-0.4227 to 0.5088	0.00303	0.8283	ns	No	18
METTL2A	0.02596	-0.4463 to 0.4869	0.0006739	0.9186	ns	No	18
TRIM25	-0.02155	-0.4836 to 0.4498	0.0004643	0.9324	ns	No	18
CLTC	0.01264	-0.4569 to 0.4767	0.0001598	0.9603	ns	No	18

PTRH2	0.01047	-0.4586 to 0.475	0.0001096	0.9671	ns	No	18
CYB561	-0.0107	-0.4888 to 0.4724	0.0001144	0.9675	ns	No	17
TLK2	-0.01001	-0.4747 to 0.459	0.0001001	0.9686	ns	No	18

Supplementary Table 6. List of antibodies used in this study

Antibody	Application	Company	Cat#	Dilution
PRR11 Antibody (aa125-360, clone OTI1A4)	Immunoblot analysis	LS Bio	LS-C336972	1:2000
PRR11 Antibody	Proximity ligation assay	LS Bio	LS-B15222	1:200
PRR11 Antibody	Immunohistochemistry	Novus	NBP1-83784	1:200
Phospho-Rb (Ser807/811) Antibody	Immunoblot analysis	Cell Signaling Technology	9308	1:1000
Rb (4H1) Mouse mAb	Immunoblot analysis	Cell Signaling Technology	9309	1:1000
Cyclin D1 (92G2) Rabbit mAb	Immunoblot analysis	Cell Signaling Technology	2978	1:1000
PI3 Kinase p110α (C73F8) Rabbit mAb	Immunoblot analysis, Co-immunoprecipitation	Cell Signaling Technology	4249	1:1000
PI3 Kinase p85α (6G10) Mouse mAb	Immunoblot analysis	Cell Signaling Technology	13666	1:1000
Anti-PI3 Kinase Antibody, p85	Proximity ligation assay	Millipore	05-212	1:200
PTEN Antibody	Immunoblot analysis	Cell Signaling Technology	9552	1:1000
Phospho-Akt (Ser473) Antibody	Immunoblot analysis	Cell Signaling Technology	9271	1:1000
AKT Antibody	Immunoblot analysis	Cell Signaling Technology	9272	1:1000
DYKDDDDK Tag (9A3) Mouse mAb	Immunoblot analysis, Co-immunoprecipitation	Cell Signaling Technology	8146	1:1000
DYKDDDDK Tag (D6W5B) Rabbit mAb (HRP Conjugate)	Immunoblot analysis, Co-immunoprecipitation	Cell Signaling Technology	86861	1:1000
HA-Tag (C29F4) Rabbit mAb	Immunoblot analysis, Co-immunoprecipitation	Cell Signaling Technology	3724	1:1000
HA-Tag (C29F4) Rabbit mAb (HRP Conjugate)	Immunoblot analysis, Co-immunoprecipitation	Cell Signaling Technology	14031	1:1000
V5-Tag (D3H8Q) Rabbit mAb	Immunoblot analysis, Co-immunoprecipitation	Cell Signaling Technology	13202	1:1000
IRS-1 (59G8) Rabbit mAb	Immunoblot analysis	Cell Signaling Technology	2390	1:1000
β-Actin (13E5) Rabbit mAb	Immunoblot analysis	Cell Signaling Technology	4970	1:4000
hFAB™ Rhodamine Anti-Actin Primary Antibody	Immunoblot analysis	BIO-RAD	12004163	1:2000
GSK-3β (27C10) Rabbit mAb	Immunoblot analysis	Cell Signaling Technology	9315	1:1000
Phospho-GSK-3β (Ser9) (5B3) Rabbit mAb	Immunoblot analysis	Cell Signaling Technology	9323	1:1000
Peroxidase AffiniPure Goat Anti-Mouse IgG	Immunoblot analysis	Jackson ImmunoResearch	115-035-146	1:3000
Peroxidase AffiniPure Goat Anti-Rabbit IgG	Immunoblot analysis	Jackson ImmunoResearch	111-035-144	1:3000
