# Supplementary material for: Proline rich 11 (PRR11) overexpression amplifies PI3K signaling and promotes antiestrogen resistance in breast cancer
Source: Nat Commun. 2020 Oct 30;11:5488. doi: 10.1038/s41467-020-19291-x (PMC7599336; doi:10.1038/s41467-020-19291-x)
Supplement: Supplementary file 3 — Description of Additional Supplementary Files [file 41467_2020_19291_MOESM3_ESM.pdf]

Description of Additional Supplementary Files

Title: Supplementary Movie 1

Description: Live cell imaging of MCF7 LTED GFP-based AKT-PH biosensor cells transfected with control siRNA. Cells were subjected to the imaging after 48 hrs from the control siRNA transfection.

Title: Supplementary Movie 2

Description: Live cell imaging of MCF7 LTED GFP-based AKT-PH biosensor cells transfected with PRR11 siRNA. Cells were subjected to the imaging after 48 hrs from the PRR11 siRNA transfection.
