# Supplementary material for: Proline rich 11 (PRR11) overexpression amplifies PI3K signaling and promotes antiestrogen resistance in breast cancer
Source: Nat Commun. 2020 Oct 30;11:5488. doi: 10.1038/s41467-020-19291-x (PMC7599336; doi:10.1038/s41467-020-19291-x)
Supplement: Supplementary file 6 — Reporting Summary [file 41467_2020_19291_MOESM6_ESM.pdf]

nature research

Corresponding author(s): Carlos L. Arteaga

Last updated by author(s): Sep 25, 2020

Reporting Summary

Nature Research wishes to improve the reproducibility of the work that we publish. This form provides structure for consistency and transparency in reporting. For further information on Nature Research policies, see our Editorial Policies and the Editorial Policy Checklist.

Statistics

For all statistical analyses, confirm that the following items are present in the figure legend, table legend, main text, or Methods section.

n/a	Confirmed	
☐	☒	The exact sample size (*n*) for each experimental group/condition, given as a discrete number and unit of measurement
☐	☒	A statement on whether measurements were taken from distinct samples or whether the same sample was measured repeatedly
☐	☒	The statistical test(s) used AND whether they are one- or two-sided<br>*Only common tests should be described solely by name; describe more complex techniques in the Methods section.*
☒	☐	A description of all covariates tested
☐	☒	A description of any assumptions or corrections, such as tests of normality and adjustment for multiple comparisons
☐	☒	A full description of the statistical parameters including central tendency (e.g. means) or other basic estimates (e.g. regression coefficient) AND variation (e.g. standard deviation) or associated estimates of uncertainty (e.g. confidence intervals)
☐	☒	For null hypothesis testing, the test statistic (e.g. *F*, *t*, *r*) with confidence intervals, effect sizes, degrees of freedom and *P* value noted<br>*Give P values as exact values whenever suitable.*
☒	☐	For Bayesian analysis, information on the choice of priors and Markov chain Monte Carlo settings
☒	☐	For hierarchical and complex designs, identification of the appropriate level for tests and full reporting of outcomes
☐	☒	Estimates of effect sizes (e.g. Cohen's *d*, Pearson's *r*), indicating how they were calculated

Our web collection on statistics for biologists contains articles on many of the points above.

Software and code

Policy information about availability of computer code

Data collection	No software was used for data collection.
Data analysis	Pearson r correlation, hazard ratio and t-tests (Nonparametric tests, two-tailed) were performed with GraphPad Prism version 8 or Microsoft Excel 2016. Gene set enrichment analysis (GSEA) was conducted with the javaGSEA interface downloaded from Broad Institute (http://software.broadinstitute.org/gsea/index.jsp). A false discovery rate (FDR) was computed using the Benjamini-Hochberg procedure with R version 3.5.2 and R studio version 1.1.463. FlowJo version 10 was used to analyze flow cytometry results. The code for 125 breast cancer-related signature is available at https://github.com/kmlee1982/Arteaga_lab.

For manuscripts utilizing custom algorithms or software that are central to the research but not yet described in published literature, software must be made available to editors and reviewers. We strongly encourage code deposition in a community repository (e.g. GitHub). See the Nature Research guidelines for submitting code & software for further information.

Data

Policy information about availability of data

All manuscripts must include a data availability statement. This statement should provide the following information, where applicable:

- Accession codes, unique identifiers, or web links for publicly available datasets
- A list of figures that have associated raw data
- A description of any restrictions on data availability

All data associated with this study are present in the paper or Supplementary information. Raw RNA sequencing and clinical data of ER+ breast tumors following long-term neoadjuvant treatment with letrozole were obtained from the previous report and are available in the Sequence Read Archive under Bioproject accession code PRJNA605185 and have been deposited in the Gene Expression Omnibus under accession code GSE145325. RNA-seq (fragments per kilobase million; FPKM) and breast tumor Ki67 data of the cohort of Miller were downloaded from supplementary information of the corresponding article. Raw RNA-seq data of the cohort

of Giltnane is obtained from the Sequence Read Archive under Bioproject accession code PRJNA272565. These published clinical cohorts are summarized in Supplementary Table 1. Somatic mutations, normalized gene expression, and clinical data in The Cancer Genome Atlas (TCGA; Cell 2015), METABRIC (Nature 2012 & Nat Commun 2016) were downloaded from cBioPortal. Copy number aberration and mutation data of metastatic breast cancers were obtained from The Metastatic Breast Cancer Project (https://www.mbcproject.org/), a project of Count Me In (https://joincountmein.org/). Gene expression, copy number, dependency and drug sensitivity data of breast cancer cell lines were downloaded through the DepMap portal (https://depmap.org/portal/). Source data are provided with this paper.

Field-specific reporting

Please select the one below that is the best fit for your research. If you are not sure, read the appropriate sections before making your selection.

[x] Life sciences [ ] Behavioural & social sciences [ ] Ecological, evolutionary & environmental sciences

For a reference copy of the document with all sections, see nature.com/documents/nr-reporting-summary-flat.pdf

Life sciences study design

All studies must disclose on these points even when the disclosure is negative.

Sample size	In general, no calculations were done to determine sample size. Sample size was determined based on standards for cell line and animal studies from our previous published studies. We have attempted to have a minimum of N=3 biological replicates with sufficient reproducibility. The number of replicates is shown in the figure legends.
Data exclusions	No data were excluded from the analyses.
Replication	All experimental findings were replicated at least 3 times with enough reproducibility. Therefore, all attempts at replication were successful.
Randomization	For all experiments, subjects were randomly assigned to experimental groups.
Blinding	In general, the investigators were blind at the time of experiment execution and data acquisition. For in vivo experiments, mice were randomized to doxycycline treatment groups after tumor established and data were collected under blinded experimental conditions.

Reporting for specific materials, systems and methods

We require information from authors about some types of materials, experimental systems and methods used in many studies. Here, indicate whether each material, system or method listed is relevant to your study. If you are not sure if a list item applies to your research, read the appropriate section before selecting a response.

Materials & experimental systems

n/a	Involved in the study	
[ ]	[x]	Antibodies
[ ]	[x]	Eukaryotic cell lines
[x]	[ ]	Palaeontology and archaeology
[ ]	[x]	Animals and other organisms
[x]	[ ]	Human research participants
[x]	[ ]	Clinical data
[x]	[ ]	Dual use research of concern

Methods

n/a	Involved in the study	
[x]	[ ]	ChIP-seq
[ ]	[x]	Flow cytometry
[x]	[ ]	MRI-based neuroimaging

Antibodies

Antibodies used	For immunoblot analyses: A PRR11 antibody was purchased from LS Bio (LS-C336972). Phospho-Rb (Ser807/811) Antibody (9308), Rb (4H1) Mouse mAb (9309), Cyclin D1 (92G2) Rabbit mAb (2978), PI3 Kinase p110α (C73F8) Rabbit mAb (4249), PI3 Kinase p85α (6G10) Mouse mAb (13666), PTEN (9552), Phospho-Akt (Ser473) Antibody (9271), AKT Antibody (9272), DYKDDDDK Tag (9A3) Mouse mAb (8146), DYKDDDDK Tag (D6W5B) Rabbit mAb (HRP Conjugate; 86861), HA-Tag (C29F4) Rabbit mAb (3724), HA-Tag (C29F4) Rabbit mAb (HRP Conjugate; 14031), IRS-1 (59G8) Rabbit mAb (2390), V5-Tag (D3H8Q) Rabbit mAb (13202), Phospho-GSK-3β (Ser9) (5B3) Rabbit mAb (9323), GSK-3β (27C10) Rabbit mAb (9315) and actin (4970) antibodies were purchased from Cell Signaling Technology. hFAB™ Rhodamine Anti-Actin primary antibody was purchased from BIO-RAD. Peroxidase AffiniPure Goat Anti-Mouse IgG (115-035-146) and Peroxidase AffiniPure Goat Anti-Rabbit IgG (111-035-144) were purchased from Jackson ImmunoResearch.<br>PRR11 (LS Bio, LS-B15222) and Anti-PI3 Kinase Antibody p85 (Millipore, 05-212) antibodies were used for proximity ligation assay. To detect HA and Flag in the co-IP experiments, DYKDDDDK Tag antibody conjugated with HRP (86861) and HA antibody conjugated with HRP (14031) were purchased from Cell Signaling Technology. For PRR11 immunohistochemistry, a PRR11 antibody was purchased from Novus (NBP1-83784).
Validation	PRR11 antibody LS-C336972 is an unconjugated mouse monoclonal antibody to PRR11 (aa125-360) from human. It is reactive with human and monkey and was validated for western blot (WB) by the manufacturer.<br>PRR11 antibody LS-B15222 is an unconjugated rabbit polyclonal antibody to PRR11 from human. It is reactive with human and mouse and was validated for ELISA and IHC (immunohistochemistry) by the manufacturer.

PRR11 Antibody NBP1-83784 was developed against Recombinant Protein corresponding to amino acids 'LAPVLLRKPSLAKALQAGPLKKDGPMQITVKDLLTVKLKKTQSLDEKRKLIPSPKARNPLVTVSDLQHVTLKPNSKVLSTR' and validated for IHC in previous reports (PMID: 26252227 and 25971332). Specificity of human PRR11 antibody verified on a Protein Array containing target protein plus 383 other non-specific proteins. Applications: WB, IHC.
Phospho-Rb (Ser807/811) (D20B12) XP® Rabbit mAb #9308 recognizes endogenous levels of Rb protein only when phosphorylated at Ser807, Ser811, or at both sites. This antibody does not cross-react with Rb phosphorylated at Ser608. Species Reactivity: Human, Monkey, Mouse, Rat. Applications: WB, IHC, immunofluorescence (IF), Flow Cytometry (FC).
Rb (4H1) Mouse mAb #9309 detects endogenous levels of total Rb protein. The antibody does not cross-react with the Rb homologues p107 or p130, or with other proteins. Species Reactivity: Bovine, Human, Monkey, Pig. Applications: WB, IP, IHC, IF.
Cyclin D1 (92G2) Rabbit mAb #2978 detects endogenous levels of total cyclin D1 protein. Species Reactivity: Human, Mouse, Rat. Applications: WB, IHC.
PI3 Kinase p110α (C73F8) Rabbit mAb #4249 detects endogenous levels of total PI3K p110α protein. Species Reactivity:Bovine, Human, Mouse, Rat. Applications: WB and IP.
PI3 Kinase p85α (6G10) Mouse mAb #13666 recognizes endogenous levels of total PI3 kinase p85α protein. This antibody does not cross-react with the PI3 kinase p85β protein. Species Reactivity: Human, Mouse. Applications: WB and IP.
Anti-PI3 Kinase Antibody, p85, N-SH3, clone AB6 #05-212 recognizes p85 alpha subunit of PI3 Kinase and does not recognize p85 beta subunit. Species reactivity: mouse, rat, human, rat, rat, human, mouse, mouse, human, human, mouse, rat. Applications: IHC, IP, WB.
PTEN Antibody #9552 detects endogenous levels of total PTEN protein. The antibody does not cross-react with related proteins. Species Reactivity: Hamster, Human, Monkey, Mouse, Rat. Applications: WB and IP.
Phospho-Akt (Ser473) Antibody #9271 detects endogenous levels of Akt1 only when phosphorylated at Ser473. This antibody also recognizes Akt2 and Akt3 when phosphorylated at the corresponding residues. It does not recognize Akt phosphorylated at other sites, nor does it recognize phosphorylated forms of related kinases such as PKC or p70 S6 kinase. Species Reactivity: Bovine, D. melanogaster, Dog, Hamster, Human, Mouse, Pig, Rat. Applications: WB, IP, IF, FC.
Akt Antibody #9272 detects endogenous levels of total Akt1, Akt2 and Akt3 proteins. The antibody does not cross-react with related kinases. Species Reactivity: Bovine, Chicken, D. melanogaster, Dog, Guinea Pig, Hamster, Human, Monkey, Mouse, Pig, Rat. Applications: WB, IP, IF, FC.
DYKDDDDK Tag (9A3) Mouse mAb #8146 detects exogenously expressed DYKDDDDK proteins in cells. The antibody recognizes the DYKDDDDK peptide, which is the same epitope recognized by Sigma's Anti-FLAG® antibodies. Applications: WB, IP, IHC, IF, FC.
DYKDDDDK Tag (D6W5B) Rabbit mAb (HRP Conjugate) #86861 is conjugated to the carbohydrate groups of horseradish peroxidase (HRP) via its amine groups. The HRP conjugated antibody is expected to exhibit the same species cross-reactivity as the unconjugated DYKDDDDK Tag (D6W5B) Rabbit mAb #14793. Applications: WB.
HA-Tag (C29F4) Rabbit mAb #3724 detects exogenously expressed proteins containing the HA epitope tag. Species Reactivity: All Species Expected. Applications: WB, IP, IHC, IF, FC.
HA-Tag (C29F4) Rabbit mAb (HRP Conjugate) #14031 is conjugated to the carbohydrate groups of horseradish peroxidase (HRP) via its amine groups. The HRP conjugated antibody is expected to exhibit the same species cross-reactivity as the unconjugated HA-Tag (C29F4) Rabbit mAb #3724. Applications: WB.
V5-Tag (D3H8Q) Rabbit mAb #13202 recognizes transfected levels of recombinant protein containing the V5 epitope tag. Species Reactivity: All Species Expected. Applications: WB, IP, IF, FC.
IRS-1 (59G8) Rabbit mAb #2390 detects endogenous levels of total IRS-1 protein. The antibody does not cross-react with IRS-2. Species Reactivity: Human, Mouse, Rat. Applications: WB.
β-Actin (13E5) Rabbit mAb #4970 detects endogenous levels of total β-actin protein. This antibody may cross-react with the γ-actin (cytoplasmic isoform). It does not cross-react with α-skeletal, α-cardiac, α-vascular smooth, or γ-enteric smooth muscle isoforms. Species Reactivity: Bovine, Human, Monkey, Mouse, Pig, Rat. Applications: WB, IHC, IF, FC.
hFAB™ Rhodamine Anti-Actin Primary Antibody #12004163, made using Human Combinatorial Antibody Library (HuCAL®) Technology, allows easy multiplexing. hFAB Rhodamine Anti-Actin IgG is optimized for use with the ChemiDoc™ MP Imaging System. Species Reactivity: Human, mouse, and rat. Applications: WB.
GSK-3β (27C10) Rabbit mAb #9315 detects endogenous levels of total GSK-3β protein. Species Reactivity: Human, Monkey, Mouse, Rat. Applications: WB, IP, IHC.
Phospho-GSK-3β (Ser9) (5B3) Rabbit mAb #9323 detects endogenous levels of GSK-3β only when phosphorylated at Ser9. The antibody may cross-react weakly with the phosphorylated form of GSK-3α due to high sequence homology. Species Reactivity: Human, Monkey, Mouse, Rat. Applications: WB, IHC, IF.
Peroxidase AffiniPure Goat Anti-Mouse IgG #115-035-146 reacts with whole molecule mouse IgG (H+L). Applications: WB, IHC, FC.
Peroxidase AffiniPure Goat Anti-Rabbit IgG #111-035-144 reacts with whole molecule rabbit IgG (H+L). Applications: WB, IHC, FC.

Eukaryotic cell lines

Policy information about cell lines

Cell line source(s)	MCF7 (ATCC® HTB-22), HCC1428 (ATCC® CRL-2327), MDA-MB-175-VII (ATCC® HTB-25), MDA-MB-134VI (ATCC® HTB-23), MDA-MB-231 (ATCC® HTB-26™), HCC38 (ATCC® CRL-2314™) and BT-474 (ATCC® HTB-20™) human breast cancer cells, HEK293 (ATCC® CRL-1573) human embryonic kidney cells and MCF10A (ATCC® CRL-10317) breast epithelial cells were purchased from ATCC. The 293FT (R70007) cells were purchased from Invitrogen in 2016.
Authentication	All cell lines were authenticated by the short-tandem repeat (STR) method.
Mycoplasma contamination	All cell lines were negative for mycoplasma contamination.
Commonly misidentified lines (See ICLAC register)	No commonly misidentified cell lines were used in the study.

Animals and other organisms

Policy information about studies involving animals; ARRIVE guidelines recommended for reporting animal research

Laboratory animals	8 weeks old female ovariectomized athymic mice (Hsd:Athymic Nude-Foxn1nu, Envigo). All mice housed in barrier facilities were maintained in individually ventilated microisolator cages. All caging equipment was autoclaved and all feed was a commercial irradiated diet. Cage manipulations and animal handling was performed in cage change stations or biosafety cabinets. Automated watering systems provided water that was purified through reverse osmosis and chlorination. The standard white light cycle was from 6:00 AM to 5:59PM and the dark cycle was from 6:00PM to 5:59AM.
Wild animals	No wild animals were used in the study.
Field-collected samples	No field collected samples were used in the study.
Ethics oversight	Animal experiments were approved by the University of Texas Southwestern Medical Center Institutional Animal Care and Use Committee.

Note that full information on the approval of the study protocol must also be provided in the manuscript.

Flow Cytometry

Plots

Confirm that:

- [ ] The axis labels state the marker and fluorochrome used (e.g. CD4-FITC).
- [ ] The axis scales are clearly visible. Include numbers along axes only for bottom left plot of group (a 'group' is an analysis of identical markers).
- [ ] All plots are contour plots with outliers or pseudocolor plots.
- [x] A numerical value for number of cells or percentage (with statistics) is provided.

Methodology

Sample preparation	For cell cycle analysis, MCF7 LTED, HCC1428 LTED, MDA-MB-134VI and MDA-MB175VII cells were fixed with 70% ethanol for 3 h at -20 centigrade. These cells were washed with PBS and then resuspended in PBS containing 100 μg/mL RNase A and 40 μg/mL propidium iodide for 10 min at room temperature.
Instrument	BD LSRFORTESSA (BD Biosciences)
Software	FlowJo Ver. 10
Cell population abundance	Since cells were originated from cell lines, cells were a homogeneous population of cancer cells.
Gating strategy	FSC/SSC gating was used to exclude debris. Then, doublet cells were excluded by height vs. area gating.

- [x] Tick this box to confirm that a figure exemplifying the gating strategy is provided in the Supplementary Information.
